# Individual resting‐state frontocingular functional connectivity predicts the intermittent theta burst stimulation response to stress in healthy female volunteers

**DOI:** 10.1002/hbm.25193

**Published:** 2020-10-03

**Authors:** Linde de Wandel, Matias M. Pulopulos, Vytautas Labanauskas, Sara de Witte, Marie‐Anne Vanderhasselt, Chris Baeken

**Affiliations:** ^1^ Department of Head and Skin Ghent University Ghent Belgium; ^2^ Ghent Experimental Psychiatry (GHEP) Lab Ghent Belgium; ^3^ Department of Experimental Clinical and Health Psychology Ghent University Ghent Belgium; ^4^ Department of Psychobiology Universidad Nacional de Educación a Distancia Madrid Spain; ^5^ Department of Psychiatry University Hospital UZ Brussel Brussels Belgium; ^6^ Department of Electrical Engineering Eindhoven University of Technology Eindhoven the Netherlands

**Keywords:** anterior cingulate cortex, cortisol, dorsolateral prefrontal cortex, functional connectivity, intermittent theta burst stimulation, Trier Social Stress Test

## Abstract

Intermittent theta burst stimulation (iTBS) delivered to the dorsolateral prefrontal cortex (DLPFC) has been investigated as a promising treatment for stress and stress‐related mental disorders such as major depression, yet large individual differences in responsiveness demand further exploration and optimization of its effectiveness. Clinical research suggests that resting‐state functional connectivity (rsFC) between the DLPFC and the anterior cingulate cortex (ACC) can predict iTBS treatment response in depression. The present study aimed to investigate whether rsFC between the left DLPFC and ACC subregions could predict the degree to which the stress system is affected by iTBS. After assessment of baseline resting‐state fMRI data, 34 healthy female participants performed the Trier Social Stress Test on two separate days, each followed by active or sham iTBS over the left DLPFC. To evaluate iTBS effects on the stress‐system, salivary cortisol was measured throughout the procedure. Our results showed that a stronger negative correlation between the left DLPFC and the caudal ACC was linked to a larger attenuation of stress‐system sensitivity during active, but not during sham iTBS. In conclusion, based on individual rsFC between left DLPFC and caudal ACC, iTBS could be optimized to more effectively attenuate deregulation of the stress system.

## Graphical abstract

The present study aimed to investigate whether resting‐state functional connectivity (rsFC) between the left dorsolateral prefrontal cortex (DLPFC) and anterior cingulate cortex (ACC) subregions could predict the degree to which the stress system is affected by intermittent theta burst stimulation (iTBS). Stronger negative correlation between the left DLPFC and the caudal ACC was linked to a larger attenuation of stress‐system sensitivity during active, but not during sham iTBS.

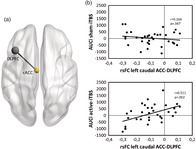

## 1 INTRODUCTION

Modern life stress is taking its toll on mental health worldwide (Hidaka, 2012). The burden of mood and stress‐related disorders on both individuals and society has been assuming alarming proportions for decades, yet relatively little progress is being made regarding treatment efficacy. Although noninvasive brain stimulation techniques such as (repetitive) transcranial magnetic stimulation (rTMS) have been showing promising effects on stress regulation and mood improvement in both neuroscientific as well as clinical contexts (Baeken et al., 2019; Blumberger et al., 2018b; Chen, Chang, Chen, & Lin, 2013; Lefaucheur et al., 2020), a growing number of studies report substantial interindividual variability in responsiveness toward TMS, as its working mechanisms are not yet fully understood (Kapur, Phillips, & Insel, 2012; López‐Alonso, Cheeran, Río‐Rodríguez, & Fernández‐del‐Olmo, 2014). Inconsistencies in responsiveness may reflect individual differences in neurophysiological processes underlying its mechanisms on the stress response. Therefore, taking individual differences into consideration is essential for expanding our insight in the working mechanisms of rTMS and possibly the further optimization of its therapeutic efficacy.

Functional connections between brain regions may prove to be important to understand TMS effects and add to heterogeneity in responsiveness. Functional connectivity between different brain areas can be quantified using resting‐state fMRI. Correlations between spontaneous fluctuations of brain activation signify a functionally connected brain network (Friston, 1994). Since rTMS treatment is technically limited to the direct stimulation of neocortical areas only—because the magnetic field decays as a function of distance, the technique often relies on connected brain regions and networks to manifest its effects. The most commonly targeted area in stress‐related psychopathology research is the dorsolateral prefrontal cortex (DLPFC). The stimulated DLPFC is thought to serve as an accessible node, projecting to the deeper limbic regions (Baeken & de Raedt, 2011; Padberg & George, 2009). Within this context, it has been proposed that resting‐state functional connectivity (rsFC) between this cortical target area and more distant regions consistently affected by stress and mood dysregulations may play an important role in the working mechanisms of rTMS, and represent a promising biomarker to explain and predict its effectiveness in stress regulation (Cash et al., 2019; Fox, Buckner, White, Greicius, & Pascual‐leone, 2012; Li, Wang, Hirvonen, Hsieh, & Bai, 2010; Weigand et al., 2018).

The most prevalent stress‐related mental health problem is major depressive disorder (MDD) (Burke, Davis, Otte, & Mohr, 2005). MDD affects a large number of brain regions and is conceived as a malfunction of brain networks rather than a single area (Li, Friston, Mody, & Hu, 2018). It comprises both cortical regions such as the lateral areas of the prefrontal cortex and limbic regions including the amygdala, hippocampus, and different parts of the anterior cingulate cortex (ACC) (Fitzgerald, Laird, Maller, & Daskalakis, 2008; Oakes, Loukas, Oskouian, & Tubbs, 2017). A handful of studies have pointed out the importance of the ACC in the prediction of TMS effectiveness, and demonstrated that the rsFC between the DLPFC and the ACC was prognostic for its therapeutic response in MDD patients (Baeken et al., 2009; Fox et al., 2012; Silverstein et al., 2015). More specifically, it was demonstrated that the effects of rTMS on depressive symptoms were most potent in patients exhibiting a greater negative correlation (anticorrelation) between the left DLPFC and the subgenual part of the ACC (sgACC) at baseline (Baeken, Marinazzo, Wu, & Van, 2014; Baeken, Vanderhasselt, et al., 2014; Fox et al., 2012). Consistently showing abnormal activation in patients with clinical depression, the sgACC indeed proves to be a successful target for several medical and neurostimulation therapies (Mayberg, 2009). Similarly, activation in the perigenual and rostral divisions of the ACC has been associated with a reduction in depressive symptoms as a result of rTMS treatment (Hernández‐ribas et al., 2013; Pizzagalli, 2011). On the other hand, the more dorsal and caudal parts of the ACC may contribute to treatment improvement as well (Fox et al., 2012; Rogers et al., 2004; Tik et al., 2017). Whereas the ventral ACC is part of a hyperactive affective network (within depression), the dorsal ACC (dACC) shows attenuated connectivity with the DLPFC as part of a disrupted cognitive control network (Li et al., 2018). Attenuated connectivity in these areas—supporting and coordinating emotion processing—may lead to a failure to control the assumed hyperactive limbic areas (Kaiser, Andrews‐Hanna, Wager, & Pizzagalli, 2019). Supporting this rationale, Fox et al. (2012) equally revealed predictive qualities of other regions, including dACC. Moreover, an exploratory study from Tik et al. (2017) investigating the effects of rTMS on a large set of resting‐state (RS) networks in a healthy sample, found that rTMS stimulation applied to the left DLPFC affected only one RS network, including the DLPFC, dACC, and medial prefrontal cortex. Even though this makes a strong case for the involvement of the ACC in rTMS depression therapy, it thus remains unclear how parts of the ACC may differentially underlie these rTMS effects, and how DLPFC–ACC connectivity relates to other important correlates of depression such as stress regulation.

Indeed, a long‐term hyperactivity of the stress system is considered one of the leading determinants of stress‐related disorders such as major depression (e.g., Burke et al., 2005; Heinze, Lin, Reniers, & Wood, 2016; Stetler & Miller, 2011; Wang et al., 2018). During stress or increased negative affect, enhanced amygdala activity causes activation of the hypothalamic–pituitary axis (HPA), cascading toward an increase of the stress hormone cortisol in the blood stream (Dedovic, Duchesne, Andrews, Engert, & Pruessner, 2009). In a healthy brain, the binding of corticotropic hormones then again leads to inhibition of the HPA‐axis, as such creating its own negative feedback‐loop (Herman, Cullinan, & Herman, 1997; Herman, Ostrander, Mueller, & Figueiredo, 2005). Sustained stress and cortisol secretion are however thought to dysregulate this feedback‐system and disrupt homeostasis. Patients with MDD frequently show disturbances in cortisol concentrations and HPA‐activation (Pariante & Lightman, 2008), ultimately affecting widespread networks in the brain including the default mode, central, executive, and salience networks (Brakowski et al., 2017). This leads to abnormal activations of these structures, causing for instance hypoactivity in ACC and DLPFC, and further weakening emotion and stress regulation (Morris, Compas, & Garber, 2012).

Of interest, it has been shown that rTMS applied to the DLPFC participates in the regulation of HPA‐activity and thereby impacts the neuroendocrine stress response, diminishing production of cortisol (Baeken, Marinazzo, et al., 2014; Baeken, Vanderhasselt, et al., 2014). Moreover, inducing sad or stressful experiences in healthy subjects was shown to achieve a pattern of brain activations consistent with the ones observed in depressed patients (Hermans, Henckens, & Joe, 2014; Ramirez‐mahaluf, Perramon, Otal, Villoslada, & Compte, 2018). The question remains nonetheless how these effects are modulated by DLPFC–ACC connectivity.

Consequently, the aim of the present sham‐controlled within‐subjects study was to investigate whether the baseline rsFC between the individual stimulation target area (the left DLPFC) and the subregions of the ACC could be predictive for rTMS efficacy in (acute) stress regulation in healthy individuals. As such, we aimed to identify a potential biomarker for rTMS‐induced stress regulation based on individual differences in neural patterns. To investigate this, we used intermittent theta burst stimulation (iTBS), an rTMS protocol that mimics endogenous theta rhythms, demonstrating similar or more potent excitatory effects than conventional high frequency stimulation (Blumberger et al., 2018a). We examined the effects of iTBS on HPA‐activity measured using cortisol concentrations. In order to observe the effects of iTBS on HPA‐axis functioning in a stressed brain, all participants were stressed using the Trier Social Stress Test (TSST) (Kirschbaum & Hellhammer, 1993).

We expected that individual differences in baseline rsFC between the DLPFC and different parts of the ACC would be predictive for HPA‐system attenuation related to active iTBS only, as indicated by a lower increase and faster recovery of cortisol levels following the TSST.

## 2 METHODS

### 2.1 Participants

Thirty‐eight healthy female volunteers in their young adulthood (aged 18–27 years old; *M* = 23.38 years old; *SD* = 3.06) were recruited through Ghent University student fora as well as social media, based on the following inclusion criteria: (a) no current/history of psychiatric disorders, as evaluated by the Mini‐International Neuropsychiatric Interview (Sheehan et al., 1998) based on DSM‐IV and ICD‐10; (b) Beck Depression Inventory (Beck, 1996, Dutch translation by van der Does, 2002) scores were below 14 points; (c) no implanted metal objects in the body; (d) no current use of psychotropic medications; (e) right handed; and (f) not pregnant. All participants were using hormonal contraceptives. Four participants were excluded for analyses either because of incomplete rsFC scans or missing cortisol data. The final sample included in the rsFC analyses was 34 participants (mean age = 23.38, *SD* = 3.06).

### 2.2 Procedure

This sham‐controlled within subject designed study was approved by the ethics committee of the Ghent University hospital. All participants gave a signed informed consent and were given a financial compensation for their participation.

The procedure took three different days to complete. First, an individual neuroanatomical MRI was collected to accurately localize the left DLPFC, followed by a resting‐state functional magnetic resonance imaging (rsfMRI) scan for the rsFC analysis. Thereafter, all participants were randomly assigned to active‐first or sham‐first stimulation. To avoid carry‐over effects between active and sham stimulation, a time delay of at least 1 week was respected. On each stimulation day, after an initial 25 min resting period, participants performed the TSST, followed by two iTBS or sham sessions with a 5 min intersession interval.

**FIGURE 1:**
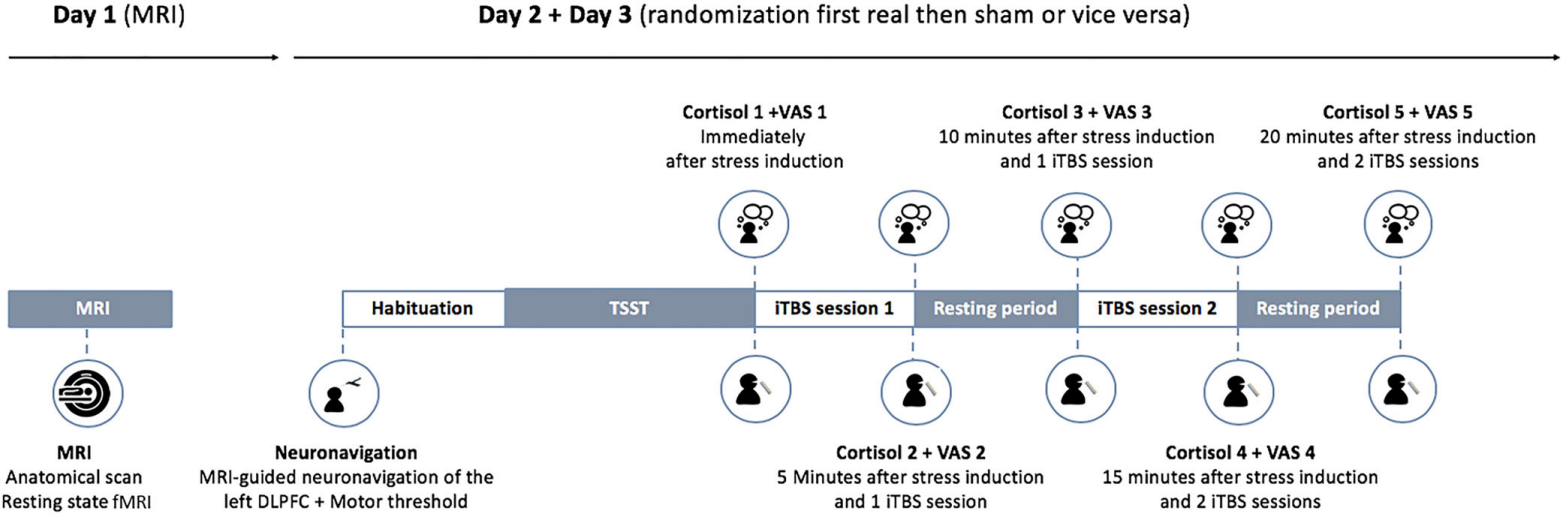
An overview of the study protocol. Abbreviations: DLPFC, dorsolateral prefrontal cortex; MRI, magnetic resonance imaging; TSST, Trier Social Stress Test; VAS, Visual Analogue Scale; iTBS, intermittent theta burst stimulation

Cortisol levels were measured immediately after the stress task, after the first iTBS/sham session, before and after the second iTBS/sham session, and 5 minutes after the second iTBS/sham session. (Although cortisol levels were also measured at the end of the habituation phase, after the preparation phase of the stress task but, for our research question, we only used the samples after the stress task for analyses.) Furthermore, to assess changes in mood, six Visual Analogue Scales (VAS; McCormack, de Horne, & Sheather, 1988) were used to detect subtle mood changes (“fatigue,” “vigor,” “anger,” “tension,” “depression,” and “cheerfulness”) during the sessions. For an overview of the study protocol, see Figure 1. Of note, the influence of psychological factors (personality, state anxiety, and rumination) on the effects of iTBS was also assessed and published in Pulopulos et al. (2019) and de Witte et al. (2020).

### 2.3 MRI acquisition parameters

A 3 T Siemens Magnetom TrioTim MRI scanner was used for the resting‐state scans on Day 1 of the protocol. First, a T1‐weighted 3D MPRAGE sequence was acquired for each participant (TR = 2,250 ms; TE = 4.18 ms; flip angle = 9°; field of view = 256 mm; 176 slices; slice thickness = 1 mm). Resting‐state functional images were acquired using a gradient echo T2*‐weighted sequence, while participants were awake and instructed to keep their eyes closed (TR = 2,500 ms; TE = 35 ms; flip angle = 80°; field of view = 224 mm; 38 slices; slice thickness = 3 mm). The resting‐state scan lasted for 7 min.

### 2.4 Stress task

To induce stress in our participants we evoked an acute stress response using the TSST (Kirschbaum & Hellhammer, 1993), and investigated the effect of active versus sham stimulation on cortisol levels after the stress task. All participants were informed they had 3 min of preparation and 5 min of speech delivery, followed by a 5 min mental arithmetic discounting task. In this variant of the TSST, the participants were positioned in front of a one‐way mirror, and they were informed that a jury was present at the other side. The jury was able to talk to the participants via the connecting sound system. The participants were told their performance was recorded with a video camera for a subsequent behavioral analysis. Previous studies using a similar version of the TSST have shown a robust stress response (e.g., Pulopulos, Baeken, & de Raedt, 2020).

### 2.5 Visual Analogue Scale

Six horizontal 10 cm VAS (McCormack et al., 1988) were used to detect changes in mood. Feelings of “fatigue,” “vigor,” “anger,” “tension,” “depression,” and “cheerfulness” were rated. The VAS subscale scores ranged from 0 to 10. Participants were asked to rate their mood at the end of the habituation phase, immediately after the TSST, after the first iTBS/sham session, right before and right after the second iTBS/sham session, and 5 min after the second iTBS/sham session.

### 2.6 Cortisol assessment

Saliva samples for cortisol assessment were collected using salivettes (Sarstedt, Germany), containing a sterile polyester swab for collecting saliva, yielding a clear and particle‐free sample. Saliva cortisol levels (μg/L) were determined by Cortisol Saliva Luminescence immunoassay (IBL International GmbH, Germany). Limit of Quantification was 0.12 μg/L and the within‐run and between‐run variation coefficients were less than 5%. The intraindividual stability of baseline salivary cortisol levels was reported to be more stable in women (Kirschbaum, Wust, & Hellhammer, 1992). To limit the influence of the circadian rhythm (Goodman, Janson, & Wolf, 2017) on the activity of the HPA axis, the sessions started after 1 p.m., and the participants performed both stimulation days at a similar time of day (there was no significant difference in the time at which participants started both sessions, *p* = .795).

### 2.7 iTBS application

Participants were randomly assigned by a computer to an active or sham‐first stimulation session. Theta burst stimulation was applied using a Magstim Rapid2 Plus1 magnetic stimulator (Magstim Company Limited, Minneapolis, MN) connected to a 70 mm “butterfly‐”shaped coil. For the sham stimulation, a specially designed sham coil was used, visually identical to the active one and producing a similar sound without active stimulation. Both active stimulation and sham coils were placed on the individually located DLPFC. To accurately target the left DLPFC, we used Brainsight neuronavigation (Brainsight, Rogue Resolutions, Inc.) to locate the center part of the left midprefrontal gyrus based on the individual anatomical MRI data. The individual resting motor threshold (110%) was determined by inducing a motor evoked potential on the right abductor pollicis brevis muscle. The following parameters were used for the iTBS sessions: 50 Hz frequency; 5 Hz burst frequency; 1,620 pulses in 54 cycles, each including 10 burst each 3 pulses with a train duration of 2 s and an intertrain interval of 6 s. Sessions (either sham or active) were separated by a 5 min resting period.

### 2.8 fMRI data preprocessing

rsfMRI images were preprocessed using CONN (Whitfield‐Gabrieli & Nieto‐Castanon, 2012; version 18a), which is an open source MATLAB based analysis toolbox for functional connectivity analysis (http://www.conn-toolbox.org). The software is powered by SPM12 (including susceptibility distortion correction, motion correction/realignment, slice‐timing correction, outlier identification, coregistration, tissue‐class segmentation, Montreal Neurological Institute [MNI] normalization, and smoothing). All functional images were first slice time corrected (interleaved, bottom‐up). Realignment parameters were estimated with respect to the first functional scan of the run. Artifact Detection Tool based outlier detection was run to identify possible outliers for first level analysis scrubbing (95% conservative parameters; z‐threshold—3.0; movement threshold—0.5). Anatomical images were coregistered and spatially normalized to the MNI template. Images were spatially smoothed with a 4 mm full‐width‐at‐half‐maximum Gaussian kernel. Linear detrending and band‐pass filtering of 0.01–0.08 Hz was applied on the blood oxygen level‐dependent (BOLD) signal to avoid low‐frequency noise and high‐frequency artifacts. White matter and cerebrospinal fluid principal components were regressed out from noise regions of interest (ROIs), in which signal is unlikely to be related to neural activity.

**FIGURE 2:**
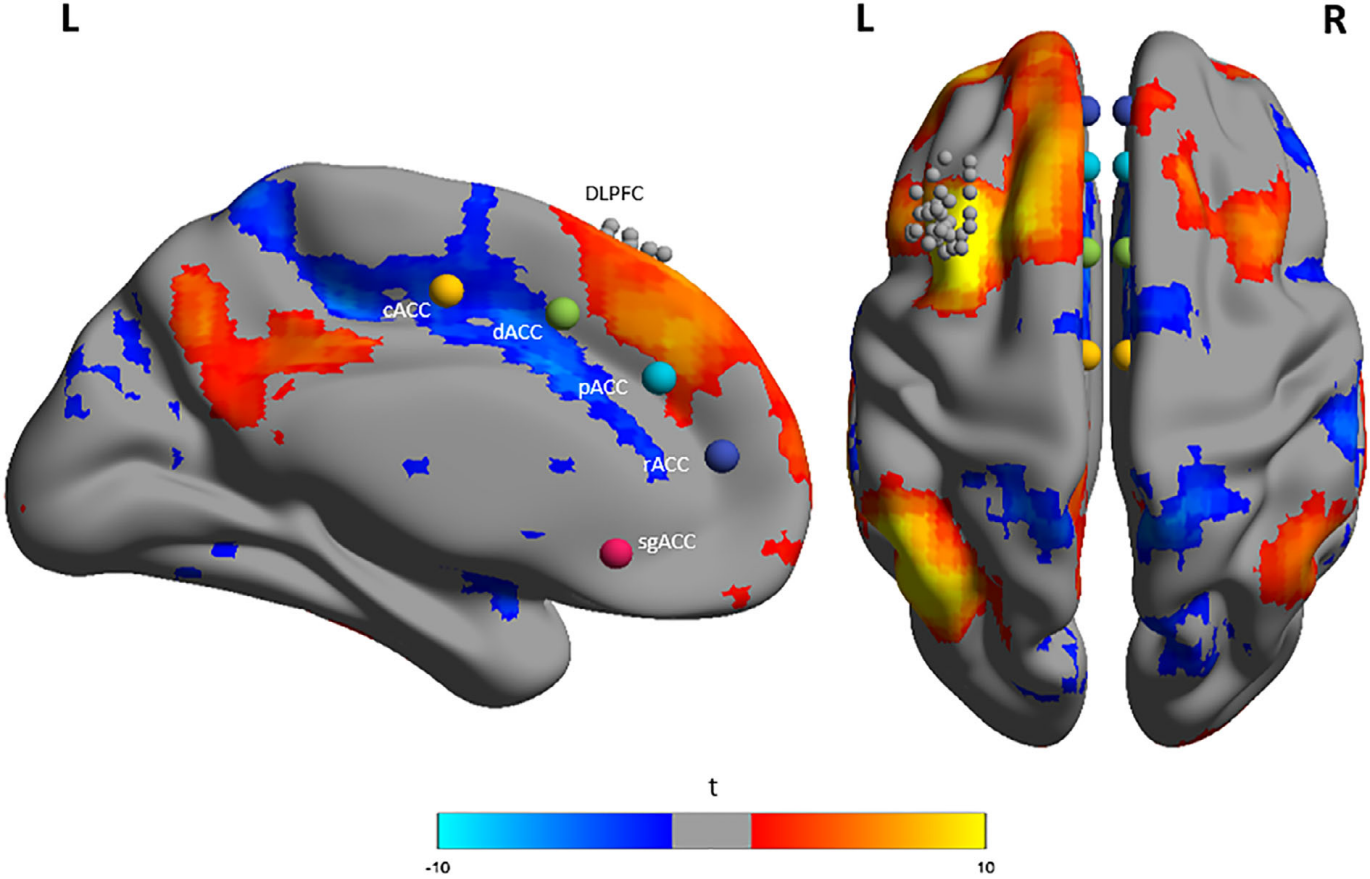
Seed‐to‐voxel functional connectivity map showing the whole‐brain correlation with individual left dorsolateral prefrontal cortex (DLPFC) seed regions in a group analysis. One‐sampled *t* tests were performed using SPM12. All analyses were corrected for multiple comparisons with the FWE option at cluster level, *p* < .001. Images made with BrainNetViewer (Xia, Wang, & He, 2013). Note: A priori defined regions of interest (ROIs) of the anterior cingulate cortex (ACC) used in the seed‐to‐seed analysis were added for visualization purposes: Caudal ACC (cACC), dorsal ACC (dACC), rostral ACC (rACC), perigenual ACC (pACC), and subgenual ACC (sgACC). Equally, the individual left DLPFC seeds are made smaller than the actual 12 mm spheres for clear visualization

First, correlation maps were obtained by extracting the BOLD time course from the individual left DLPFC seed regions, then computing the correlation coefficients characterizing the correlations between that time course and the time courses from all other brain voxels. These correlation maps were submitted to a random‐effects analysis in SPM12. A one‐sampled *t* test was performed, corrected for multiple comparisons with the FWE option at cluster level, *p* < .001 (see Figure 2).

**FIGURE 3:**
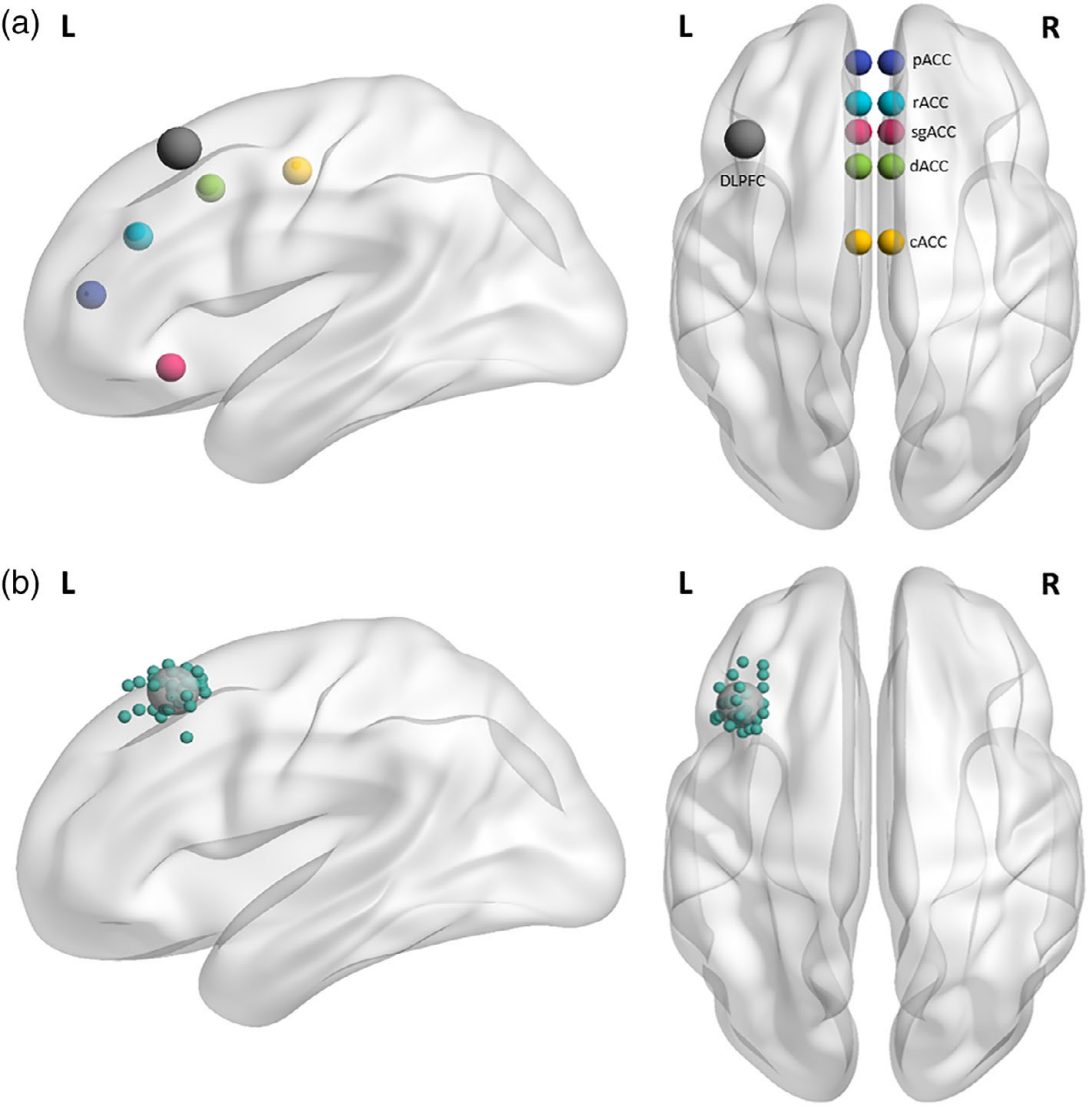
(a) Overview of regions of interest included in the analyses. Note: Coordinates for the anterior cingulate cortex (ACC) regions were derived from Kelly et al. (2009): Caudal ACC (cACC), dorsal ACC (dACC), rostral ACC (rACC), perigenual ACC (pACC), and subgenual ACC (sgACC). Functional connectivity indices were calculated from each ACC region of interest (ROI) with the individual stimulation sites on the left dorsolateral prefrontal cortex (DLPFC). The mean of these individual sites is depicted here for visualization purposes but is not used in analyses. (b) Depiction of all individual stimulation regions used for the analyses. Note: Images made with BrainNetViewer (Xia et al., 2013). The individual left DLPFC seeds are made smaller than the actual 12 mm spheres for clear visualization

Second, to answer our main research question, ROIs were defined as spheres of different radii using the CONN interface. The left DLPFC ROI was defined according to the individual stimulation areas detected with neuronavigation, as 12 mm radii spheres. The coordinates of five left and right ACC structures (subgenual, caudal, dorsal, rostral, perigenual) were defined according to Kelly et al. (2009), as 6 mm spheres (see Figure 3).

### 2.9 Statistical analyses

To examine whether the stress task provoked a similar psychological stress response during both sessions, mood changes were analyzed using a mixed MANOVA, with stimulation (active vs. sham stimulation) and time (T_1_, T_2_, T_3_, T_4_, and T_5_) as the within‐subject factors, Order (active‐first vs. sham‐first) as the between‐subjects factor, and the six VAS mood scales (“fatigue,” “vigor,” “anger,” “tension,” “depression,” and “cheerfulness”) as the multiple dependent variables (positive mood scales were reversed). Higher scores indicate more negative affect.

Cortisol levels were log transformed because they did not show normal distributions. Following the formulas proposed by Pruessner, Kirschbaum, Meinlschmid, and Hellhammer (2003), we computed the area under the curve with respect to the increase (*AUCi*), as an index of the sensitivity of the HPA‐axis to the stressful event, and the area under the curve with respect to the ground (*AUCg*), as an index of the total cortisol release by the HPA‐axis and reflecting the intensity of the HPA‐axis response (Fekedulegn et al., 2007; Pruessner et al., 2003). Because in this study, we are specifically interested in the influence of rsFC on the effects of iTBS on the HPA axis activity in a stressed subject, the *AUCi* and *AUCg* were calculated using the five salivary samples collected after the stress task. Importantly, we also investigated the change in cortisol levels from baseline to the cortisol peak after the stress task, to assess whether the stress task provoked a significant increase in participants' cortisol levels. Paired *t* tests show that the increase in cortisol levels was statistically significant for the active (*M* = 0.34, *SD* = 0.72; *t*(33) = −2.47, *p* = .018) and the sham group (*M* = 0.40, *SD* = 0.94; *t*(33) = −2.73, *p* = .010). Moreover, paired t‐tests showed that there were no significant differences between the two stimulation days in cortisol levels at baseline, immediately before the stress task (i.e., after the preparation phase), and immediately after the stress task (before the first iTBS/sham session), regardless of stimulation type (*p* > .459). The statistical conclusions are the same if the analyses are performed controlling for the change in cortisol levels from baseline to immediately after the stress task.

We performed two mixed ANOVAs to investigate the effects of iTBS on the *AUCi* and *AUCg* indexes. The active and sham *AUCi* and *AUCg* values were used as the dependent variables. As a within‐subjects factor we included Stimulation (active‐iTBS vs. sham‐iTBS), and as a between‐subjects factor, we included Order (first session active vs. first session sham). In a second step, to investigate the influence of the rsFC between ACC and left DLPFC on the effects of iTBS on cortisol secretion, we used mixed ANCOVAs with *AUCi* and *AUCg* values as dependent variables, stimulation (active‐iTBS vs. sham‐iTBS) as the within‐subject factor, order (first session active vs. first session sham) as a the between‐subjects factor, and the rsFC indexes as covariates. Independent analyses were performed for each rsFC index (i.e., left cACC‐DLPFC, right cACC‐DLPFC, left dACC, right dACC‐DLPFC, left rACC‐DLPFC, right rACC‐DLPFC, left pACC‐DLPFC, right pACC‐DLPFC, left sgACC‐DLPFC, right sgACC‐DLPFC). Given the number of analyses performed to investigate the influence of rsFC on the effect of iTBS on *AUCi* and *AUCg*, we applied a Bonferroni correction to the *p*‐value of the mixed ANCOVAs in order to avoid Type I error. Therefore, the significance level was set at *p* = .0025 (0.05 divided by 20 comparisons), two‐tailed. When analyses showed a statistically significant interaction between stimulation (active‐iTBS vs. sham‐iTBS) and the rsFC indexes, correlations were used to investigate the relationship between *AUCg* and *AUCi* for the sham and active iTBS sessions and the rsFC indexes. Importantly, the time of day when the experiment is performed may affect the stress‐induced changes in HPA axis activity (for a meta‐analysis see Goodman et al., 2017). The statistical conclusions of this study remain unaltered if the time of day when the stress task was performed is included as a covariate in the analyses (results are presented in supplementary materials). We screened our data for univariate and multivariate outliers (|z| ≥ 3 *SD*). No outliers were found in this study. All the analyses were performed using SPSS 24.0 (IBM SPSS Statistics 24.0).

## 3 RESULTS

The final sample included in the rsFC analyses was 34 participants. For rsFC and cortisol values of the final sample included in the analyses (*n* = 34), we refer to Table 1. For the mood analyses, one more participant was excluded on the basis of missing data. For VAS values of the final sample included in the analyses (*n* = 33), we refer to Table 2.

**TABLE 1:** Mean ratings and *SD* for AUCi, AUCg, and rsFC values of the entire sample (*n* = 34)

		*M* (*SD*)
AUCi	Active	149.97 (681.97)
	Sham	29.94 (529.25)
AUCg	Active	−252.01 (1,803.03)
	Sham	−230.73 (1,558.72)
rsFC right ACC	cACC	−0.09 (0.20)
	dACC	−0.18 (0.26)
	rACC	−0.09 (0.22)
	pACC	0.06 (0.24)
	sgACC	0.01 (0.23)
rsFC left ACC	cACC	−0.10 (0.12)
	dACC	−0.11 (0.27)
	rACC	0.13 (0.25)
	pACC	0.09 (0.31)
	sgACC	−0.02 (0.25)

Abbreviations: ACC, anterior cingulate cortex; AUCi, the area under the curve with respect to increase; AUCg, area under the curve with respect to ground; cACC, caudal ACC; dACC, dorsal ACC; rACC, rostral ACC; rsFC, resting‐state functional connectivity; pACC, perigenual ACC; sgACC, subgenual ACC.

**TABLE 2:** Mean ratings and *SD* for the VAS measures throughout the protocol (also see Figure 1)

		VAS *M* (*SD*)					
Time	iTBS	Fatigue	Vigor	Anger	Tension	Depression	Cheerfulness
T1	Active	3.56 (2.24)	5.81 (2.17)	0.73 (1.01)	2.34 (2.01)	0.31 (0.46)	6.20 (1.95)
	Sham	3.54 (2.47)	6.04 (2.00)	0.83 (1.24)	2.35 (2.15)	0.23 (0.25)	6.33 (2.19)
T2	Active	3.85 (2.11)	5.57 (2.26)	0.59 (1.16)	1.67 (1.81)	0.30 (0.34)	6.03 (2.10)
	Sham	3.76 (2.10)	5.55 (2.21)	0.38 (0.41)	1.60 (1.94)	0.24 (0.27)	6.46 (1.88)
T3	Active	3.83 (2.17)	5.56 (2.22)	0.66 (1.61)	1.31 (1.47)	0.21 (0.20)	6.07 (1.94)
	Sham	3.40 (2.25)	5.70 (2.08)	0.61 (0.97)	1.47 (1.97)	0.47 (1.31)	6.20 (2.14)
T4	Active	3.65 (2.18)	5.54 (2.31)	0.32 (0.48)	1.45 (1.90)	0.25 (0.25)	6.21 (2.10)
	Sham	4.22 (2.60)	5.48 (2.48)	0.43 (0.60)	1.31 (1.98)	0.38 (1.27)	6.29 (2.34)
T5	Active	3.82 (2.18)	5.97 (2.24)	0.34 (0.40)	1.05 (1.43)	0.23 (0.29)	6.02 (2.48)
	Sham	4.03 (2.60)	5.95 (2.44)	0.57 (1.15)	1.00 (1.84)	0.40 (1.27)	6.15 (2.42)

*Note:* Scores are expressed on scales from 0 to 10 cm ranging from absence of the emotion to the max of the emotion. *N* = 33.

Abbreviations: iTBS, intermittent theta burst stimulation; VAS, Visual Analogue Scale.

### 3.1 Mood

Mixed MANOVA revealed a significant main effect of Time (*F*(24,488) = 3.55, *p* < .001) and a significant interaction between stimulation and order (*F*(6,26) = 3.52, *p* = .011). There was no significant main effect of stimulation type (*F*(6,26) = .46, *p* = .834) or interaction between stimulation and time (*F*(24,488) = 1.06, *p* = .390). Performing univariate analyses of variance (ANOVAs) on the subscales of VAS results, we found no significant effect of time on “fatigue” (*F*(4,124) = 2.08, *p* = .088), “depression” (*F*(4,124) = .11, *p* = .979) or “cheerfulness” (*F*(4,124) = .51, *p* = .727). There was a significant main effect on “tension” (*F*(4,124) = 12.78, *p* < .001) and “anger” (*F*(4,124) = 3.14, *p* = .017) as it increased during the experiment, while “vigor” (*F*(4,124) = 2.88, *p* = .026) decreased. We only found a significant interaction effect between stimulation and order on “anger” (*F*(1,31) = 5.35, *p* = .028) and “tension” (*F*(1,31) = 5.78, *p* = .022). Although anger and tension generally increased during the protocol, participants who received active stimulation in the second session were angrier and more tense compared to participants who received sham stimulation. This effect could not be found when the active stimulation was given during the first session.

### 3.2 Left DLPFC seed‐to‐voxel correlation connectivity maps

Concerning our predefined ACC ROIs, we found that at the group level, the left DLPFC seeds to whole brain voxel connectivity overlapped in particular with the left caudal ACC seed showing overall a negative correlation (see Figure 2).

### 3.3 Effects of iTBS on cortisol

#### 3.3.1 Direct effects of iTBS on the activity of the HPA axis

The results of the mixed ANOVA with *AUCi* and *AUCg* as dependent variables showed no significant main effect of Stimulation (*AUCi*: *F*(1,32) = 0.95, *p* = .337; *AUCg*: *F*(1,32) = 0.03, *p* = .871), Order (*AUCi*: *F*(1,32) = 0.49, *p* = .487; *AUCg*: *F*(1,32) = 0.68, *p* = .415), or the interaction between Stimulation and Order (*AUCi*: *F*(1,32) = 1.10, *p* = .302; *AUCg*: *F*(1,32) = 0.45, *p* = .508). These results indicate that iTBS does not affect *AUCi* and *AUCg* after being stressed.

#### 3.3.2 Influence of rsFC on the effects of iTBS on the activity of the HPA axis

Independent mixed ANCOVAs with each rsFC index as a covariate were performed to investigate the influence of rsFC between the DLPFC and the subparts of ACC on the effects of iTBS on the activity of the HPA axis after stress. We observed no significant main effect of Stimulation and Order for all the analyses with *AUCi* (*F*(1,31) < 1.25, *p* > .272) and *AUCg* (*F*(1,31) < 0.73, *p* > .400). Moreover, none of the rsFC indexes showed a significant main effect (*AUCi*: *F*(1,31) < 2.72, *p* > .109; *AUCg*: *F*(1,31) < 0.50, *p* > .484).

Regarding the influence of the rsFC on the effects of iTBS on the stress response, we observed a significant rsFC interaction between the left DLPFC and the left cACC and the factor Stimulation for *AUCi* (*F*(1,31) = 12.52, *p* = .001), but not for *AUCg* (*F*(1,31) = 4.23, *p* = .048). None of the other interactions between rsFC indexes and Stimulation showed a significant effect (*AUCi*: *F*(1,31) < 1.56, *p* > .221; *AUCg*: *F*(1,31) < 2.92, *p* > .098).

**FIGURE 4:**
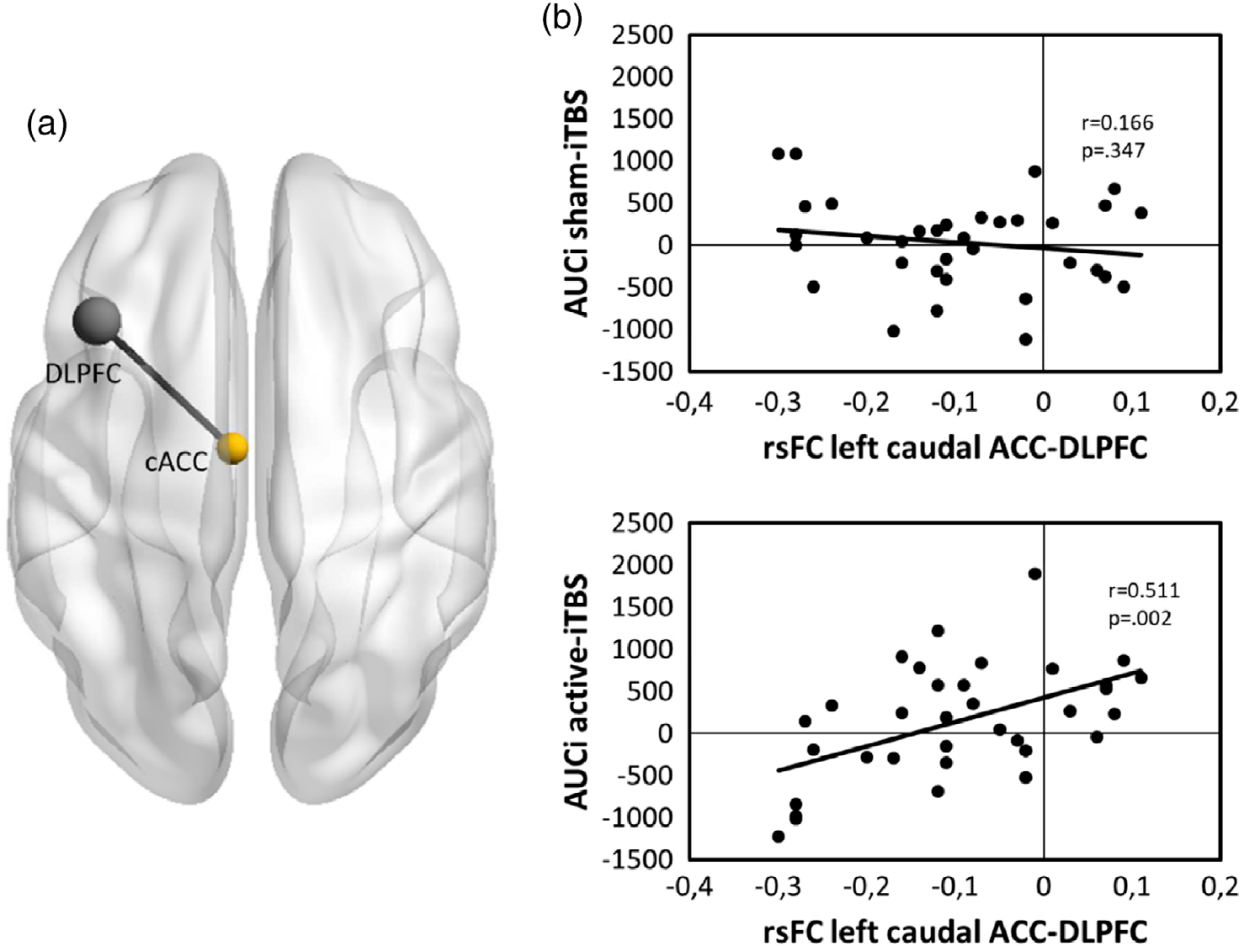
(a) Resting‐state functional connectivity (rsFC) predictive for intermittent theta burst stimulation (iTBS) effects on cortisol area under the curve with respect to increase (*AUCi*). Image made with BrainNetViewer (Xia et al., 2013). (b) Scatterplots for the rsFC associations between the left caudal anterior cingulate cortex (cACC) and the left DLPFC and the *AUCi* during sham (left panel) and active (right panel) iTBS

Finally, correlation analyses were performed to further investigate the meaning of the significant interaction between Stimulation and the rsFC between the left DLPFC and the left cACC. The results of the correlation analysis showed a significant rsFC association between left cACC‐DLPFC and the *AUCi* during the active iTBS session (*r* = .511, *p* = .002), but not during the sham iTBS session (*r* = −.166, *p* = .347) (Figure 3). These results indicate that the weaker the rsFC between the left DLPFC and the left cACC, the lower the *AUCi* during active‐iTBS, but not during sham‐iTBS (see Figure 4).

## 4 DISCUSSION

The present study is the first to investigate how baseline rsFC between the individual stimulation site on the left DLPFC and distinct parts of the ACC may predict the effects of iTBS on stress reactivity in healthy subjects. Concerning the baseline group functional connectivity analysis, we observed a negative correlation between the individual left DLPFC targets and the more dorsal parts of the ACC. In general, active and sham stimulation did not affect the stress response differently. Only when considering individual differences in rsFC strengths, and with active iTBS only, we found that stronger baseline rsFC anticorrelations between the individual stimulation site (left DLPFC) and the left cACC showed predictive value for lower cortisol levels (*AUCi*) after stress. These observations suggest that the incorporation of individual brain biomarkers may be of essence to optimize rTMS effectiveness in terms of stress regulation and increase its response rate.

The dorsal and caudal components of the ACC are known to have strong connections with the DLPFC. This more “cognitive” subdivision of the ACC is part of a larger cognitive control network—including the DLPFC—that plays a central role in the neurobiology of depression (Li et al., 2018; Pizzagalli, 2011; Wang, Yang, Sun, Shi, & Duan, 2016) and takes part in emotion and stress regulation (de Raedt & Hooley, 2016). In line with this, the cACC has been linked to defense preparation, and activates as a result of negative social feedback (Büchel et al., 2002; Rijpkema, Smidts, Klucharev, & Hyto, 2009). The latter is also an important component of our paradigm used to evoke acute stress and manipulate HPA‐axis activity. The TSST has indeed been found to activate multiple parts of the ACC including the cACC, as well as parts of the HPA‐axis (Dedovic, Aguiar, & Pruessner, 2009). The connectivity between the stimulated DLPFC and cACC might therefore be essential to stress reactivity and regulation. Our results imply that a stronger anticorrelated rsFC between the left DLPFC and left cACC in healthy individuals can be predictive for more effectiveness of iTBS in the regulation of HPA‐axis activity. A whole‐brain meta‐analysis of Hamilton et al. (2012) similarly described a dissociation between these regions by revealing that when confronted with negative stimuli, depressed individuals showed greater amygdala and ACC activation, while the DLPFC showed an attenuated response compared to healthy individuals. In line with this, several studies reported reduced left DLPFC activation linked to psychobiological stress levels and anxiety, while increased activation in more dorsal parts of the ACC was found to be related to threat (Balderston et al., 2017; Qin, Hermans, van Marle, Luo, & Fernández, 2009). Furthermore, Seeley et al. (2007) showed that a stronger ACC connectivity with a salience network responding to personally relevant information, was linked with greater anticipatory anxiety before the experiment. Since the experimental setting, the stressor and the successive stress‐recovery period serve as a negative or stressful context and set these specific regions and networks into action, iTBS might show more effectiveness in individuals who are at baseline more sensitive to negative information and experiences. Although they did not look at the effects on the HPA‐axis specifically, in line with our results Klooster et al. (2019) linked structural connectivity between the patient‐specific stimulation site in the left DLPFC and the caudal and posterior parts of the ACC with clinical response to accelerated iTBS in depressed patients. Contrary to what we might have expected based on previous literature, baseline rsFC with the sgACC was not found to be predictive for iTBS outcome on the stress system. Being part of an emotional network influenced by fluctuations in mood states it is possible that the left DLPFC‐sgACC connections are not stable enough to be used as predictor for mood and stress changes induced by iTBS in a nondepressed sample. The absence of a pre‐existing hyperconnectivity between these regions in healthy subjects might limit the range to which this connection and these regions can be altered by iTBS. Indeed, in a clinically depressed sample the sgACC is found to be more continuously hyperactive and could be a more reliable biomarker in MDD patients (Disner 2011; Fox et al., 2012; Fox, Liu, & Pascual‐leone, 2013).

Although this study has several important strengths, such as the use of a within‐subjects design where each participant receives both sham and active stimulation, as well as the use of a well‐validated stressor, it should be noted that our study also has several limitations. A first limitation entails the timing of our stimulation. So close to the stressor, iTBS stimulation might have affected both the peak of stress as well as the recovery speed, making it more difficult to disentangle these effects. Second, we also used a 5‐min interval in between the two iTBS sessions, contrasting with most iTBS protocols using a 10–15 min interval. Third, since we only included female subjects in our study, our results cannot be simply generalized to men as gender differences are documented in response to the TSST (Kelly, Tyrka, Anderson, Price, & Carpenter, 2008; Kirschbaum et al., 1992). A fourth limitation entails the fact that we did not include a behavioral measure of subjective stress experience, notwithstanding that the VAS subscale tension scores significantly increased after being stressed by the TSST. We can only rely on the cortisol values to assess the effectiveness of the TSST in our sample. Finally, it could be considered that the use of hormonal contraceptives could have interfered with our results, as differences in menstrual cycle phase (active vs. inactive) might have affected cortisol responses. However, although our within‐subjects design does indeed not negate the issue of hormonal variation unless participants would be assessed in the same stage of their menstrual cycle for each testing session, a recent study found no difference in stress response between women in the active versus inactive phase (Ycaza, Faude, Nielsen, Locke, & Mather, 2019).

In conclusion, the results of this study highlight the importance of considering individual differences in rsFC in order to optimize iTBS effectiveness, and to potentially increase treatment response rate for stress‐related disorders. Neurostimulation based upon rsFC between the DLPFC and cACC might prove to be a more effective method to attenuate the HPA‐axis deregulation. Nevertheless, future research is needed to further elucidate the effects of iTBS on the HPA‐axis in depressed individuals, considering rsFC parameters.

## Supporting information


**Appendix** **S1:** Supplementary materials

## Data Availability

Data available on request.

## References

[hbm25193-bib-0001] Baeken, C. , Brem, A. , Arns, M. , Ganho‐a, A. , Brunoni, A. R. , & Filipc, I. (2019). Repetitive transcranial magnetic stimulation treatment for depressive disorders: Current knowledge and future directions. Current Opinion in Psychiatry, 32(5), 409–415. 10.1097/YCO.0000000000000533 31145145 PMC6688778

[hbm25193-bib-0002] Baeken, C. , & de Raedt, R. (2011). Neurobiological mechanisms of repetitive transcranial magnetic stimulation on the underlying neurocircuitry in unipolar depression. Dialogues in Clinical Neuroscience, 13(1), 140–146. 10.31887/DCNS.2011.13.1/cbaeken PMC3181968 21485753

[hbm25193-bib-0003] Baeken, C. , de Raedt, R. , van Hove, C. , Clerinx, P. , de Mey, J. , & Bossuyt, A. (2009). Hf‐RTMS treatment in medication‐resistant melancholic depression: Results from 18FDG‐PET brain imaging. CNS Spectrums, 14(8), 439–448. 19890238 10.1017/s1092852900020411

[hbm25193-bib-0004] Baeken, C. , Marinazzo, D. , Wu, G. , & Van, P. (2014). Accelerated HF‐rTMS in treatment‐resistant unipolar depression: Insights from subgenual anterior cingulate functional connectivity. World Journal of Biological Psychiatry, 32(4), 286–297. 10.3109/15622975.2013.872295 24447053

[hbm25193-bib-0005] Baeken, C. , Vanderhasselt, M. A. , Remue, J. , Rossi, V. , Schiettecatte, J. , & Anckaert, E. (2014). One left dorsolateral prefrontal cortical HF‐rTMS session attenuates HPA‐system sensitivity to critical feedback in healthy females. Neuropsychologia, 57, 112–121. 10.1016/j.neuropsychologia.2014.02.019 24593899

[hbm25193-bib-0006] Balderston, N. L. , Vytal, K. E. , Connell, K. O. , Torrisi, S. , Letkiewicz, A. , Ernst, M. , & Grillon, C. (2017). Anxiety patients show reduced working memory related DLPFC activation during safety and threat. Depression and Anxiety, 36, 25–36. 10.1002/da.22518 PMC5079837 27110997

[hbm25193-bib-0007] Beck, A. T. (1996). Comparison of Beck Depression Inventories ‐IA and ‐II in psychiatric outpatients. Journal of Personality Assessment, 67(3), 588–597. 8991972 10.1207/s15327752jpa6703_13

[hbm25193-bib-0008] Blumberger, D. M. , Vila‐rodriguez, F. , Thorpe, K. E. , Feffer, K. , Noda, Y. , Giacobbe, P. , … Downar, J. (2018a). Articles effectiveness of theta burst versus high‐frequency repetitive transcranial magnetic stimulation in patients with depression (THREE‐D): A randomised non‐inferiority trial. The Lancet, 391(10131), 1683–1692. 10.1016/S0140-6736(18)30295-2 29726344

[hbm25193-bib-0009] Blumberger, D. M. , Vila‐rodriguez, F. , Thorpe, K. E. , Feffer, K. , Noda, Y. , Giacobbe, P. , … Downar, J. (2018b). Effectiveness of theta burst versus high‐frequency repetitive transcranial magnetic stimulation in patients with depression (THREE‐D): a randomised non‐inferiority trial. The Lancet, 391(10131), 1683–1692. 10.1016/S0140-6736(18)30295-2 29726344

[hbm25193-bib-0010] Brakowski, J. , Spinelli, S. , Nadja, D. , Bosch, O. G. , Manoliu, A. , Holtforth, M. G. , & Seifritz, E. (2017). Resting state brain network function in major depression—Depression symptomatology, antidepressant treatment effects, future research. Journal of Psychiatric Research, 92(2017), 147–159. 10.1016/j.jpsychires.2017.04.007 28458140

[hbm25193-bib-0011] Büchel, C. , Bornhövd, K. , Quante, M. , Glauche, V. , Bromm, B. , & Weiller, C. (2002). Dissociable neural responses related to pain intensity, stimulus intensity, and stimulus awareness within the anterior cingulate cortex: A parametric single‐trial laser functional magnetic resonance imaging study. Journal of Neuroscience, 22(3), 970–976. 11826125 10.1523/JNEUROSCI.22-03-00970.2002 PMC6758484

[hbm25193-bib-0012] Burke, H. M. , Davis, M. C. , Otte, C. , & Mohr, D. C. (2005). Depression and cortisol responses to psychological stress: A meta‐analysis. Psychoneuroendocrinology, 30, 846–856. 10.1016/j.psyneuen.2005.02.010 15961250

[hbm25193-bib-0013] Cash, R. F. H. , Zalesky, A. , Thomson, R. H. , Tian, Y. , Cocchi, L. , & Fitzgerald, P. B. (2019). Subgenual functional connectivity predicts antidepressant treatment response to transcranial magnetic stimulation: Independent validation and evaluation of personalization. Biological Psychiatry, 86, e5–e7. 10.1016/j.biopsych.2018.12.002 30670304

[hbm25193-bib-0014] Chen, S.‐J. , Chang, C.‐H. , Chen, S.‐T. , & Lin, C. C. H. (2013). Superior antidepressant effect occurring 1 month after rTMS: Add‐on rTMS for subjects with medication‐resistant depression. Neuropsychiatric Disease and Treatment, 2013(9), 397–401. 10.2147/NDT.S40466 PMC3617929 23576870

[hbm25193-bib-0067] Clare Kelly, A. M. , Di Martino, A. , Uddin, L. Q. , Shehzad, Z. , Gee, D. G. , Reiss, P. T. , & Milham, M. P. (2009). Development of anterior cingulate functional connectivity from late childhood to early adulthood. Cerebral Cortex, 19(3), 640–657. 10.1093/cercor/bhn117 18653667

[hbm25193-bib-0015] de Raedt, R. , & Hooley, J. M. (2016). The role of expectancy and proactive control in stress regulation: A neurocognitive framework for regulation expectation. Clinical Psychology Review, 45, 45–55. 10.1016/j.cpr.2016.03.005 27064551

[hbm25193-bib-0016] de Witte, S. , Baeken, C. , Pulopulos, M. M. , Josephy, H. , Schiettecatte, J. , Anckaert, E. , … Vanderhasselt, M. (2020). The effect of neurostimulation applied to the left dorsolateral prefrontal cortex on post‐stress adaptation as a function of depressive brooding. Progress in Neuropsychopharmacology and Biological Psychiatry, 96, 109687 10.1016/j.pnpbp.2019.109687 31356848

[hbm25193-bib-0018] Dedovic, K. , Aguiar, C. D. , & Pruessner, J. C. (2009). What stress does to your brain: A review of neuroimaging studies. Canadian Journal of Psychiatry, 54(1), 6–15. 19175975 10.1177/070674370905400104

[hbm25193-bib-0019] Dedovic, K. , Duchesne, A. , Andrews, J. , Engert, V. , & Pruessner, J. C. (2009). The brain and the stress axis: The neural correlates of cortisol regulation in response to stress. NeuroImage, 47(3), 864–871. 10.1016/j.neuroimage.2009.05.074 19500680

[hbm25193-bib-0070] Disner, S. G. , Beevers, C. G. , Haigh, E. A. P. , & Beck, A. T. (2011). Neural mechanisms of the cognitive model of depression. Neuroscience, 12(8), 467–477. 10.1038/nrn3027 21731066

[hbm25193-bib-0020] Fekedulegn, D. B. , Andrew, M. E. , Burchfiel, C. M. , Violanti, J. M. , Hartley, T. A. , Charles, L. E. , & Miller, D. B. (2007). Area under the curve and other summary indicators of repeated waking cortisol measurements. Psychosomatic Medicine, 2007(69), 651–659. 10.1097/PSY.0b013e31814c405c 17766693

[hbm25193-bib-0021] Fitzgerald, P. B. , Laird, A. R. , Maller, J. , & Daskalakis, Z. J. (2008). A meta‐analytic study of changes in brain activation in depression. Human Brain Mapping, 695(June 2007), 683–695. 10.1002/hbm.20426 PMC2873772 17598168

[hbm25193-bib-0022] Fox, M. D. , Buckner, R. L. , White, M. P. , Greicius, M. D. , & Pascual‐leone, A. (2012). Efficacy of TMS targets for depression is related to intrinsic functional connectivity with the subgenual cingulate. Biological Psychiatry, 72(7), 595–603. 10.1016/j.biopsych.2012.04.028.Efficacy 22658708 PMC4120275

[hbm25193-bib-0023] Fox, M. D. , Liu, H. , & Pascual‐leone, A. (2013). Identification of reproducible individualized targets for treatment of depression with TMS based on intrinsic connectivity. NeuroImage, 66, 151–160. 10.1016/j.neuroimage.2012.10.082 23142067 PMC3594474

[hbm25193-bib-0024] Friston, K. J. (1994). Functional and effective connectivity in neuroimaging: A synthesis. Human Brain Mapping, 2, 56–78.

[hbm25193-bib-0025] Goodman, W. K. , Janson, J. , & Wolf, J. M. (2017). Psychoneuroendocrinology meta‐analytical assessment of the effects of protocol variations on cortisol responses to the Trier Social Stress Test. Psychoneuroendocrinology, 80, 26–35. 10.1016/j.psyneuen.2017.02.030 28292684

[hbm25193-bib-0026] Hamilton, J. P. , Ph, D. , Lemus, M. G. , Johnson, R. F. , Gotlib, I. H. , & Ph, D. (2012). Functional neuroimaging of major depressive disorder: A meta‐analysis and new integration of baseline activation and neural response data. American Journal of Psychiatry, 169(7), 693–703. 22535198 10.1176/appi.ajp.2012.11071105 PMC11889638

[hbm25193-bib-0027] Heinze, K. , Lin, A. , Reniers, R. L. E. P. , & Wood, S. J. (2016). Longer‐term increased cortisol levels in young people with mental health problems. Psychiatry Research, 236, 98–104. 10.1016/j.psychres.2015.12.025 26749569 PMC4756272

[hbm25193-bib-0028] Herman, J. P. , Cullinan, W. E. , & Herman, J. P. (1997). Neurocircuitry of stress: Central control of the hypothalamo–pituitary–adrenocortical axis. Trends in Neurosciences, 20, 78–84. 9023876 10.1016/s0166-2236(96)10069-2

[hbm25193-bib-0029] Herman, J. P. , Ostrander, M. M. , Mueller, N. K. , & Figueiredo, H. (2005). Limbic system mechanisms of stress regulation: Hypothalamo‐pituitary‐adrenocortical axis. Progress in Neuro‐Psychopharmacology & Biological Psychiatry, 29(2005), 1201–1213. 10.1016/j.pnpbp.2005.08.006 16271821

[hbm25193-bib-0030] Hermans, E. J. , Henckens, M. J. A. G. , & Joe, M. (2014). Dynamic adaptation of large‐scale brain networks in response to acute stressors. Trends in Neurosciences, 37(6), 304–314. 10.1016/j.tins.2014.03.006 24766931

[hbm25193-bib-0031] Hernández‐ribas, R. , Deus, J. , Pujol, J. , Segalàs, C. , Vallejo, J. , Menchón, J. M. , … Soriano‐mas, C. (2013). Identifying brain imaging correlates of clinical response to repetitive transcranial magnetic stimulation (rTMS) in major depression. Brain Stimulation, 6(1), 54–61. 10.1016/j.brs.2012.01.001 22417767

[hbm25193-bib-0032] Hidaka, B. H. (2012). Depression as a disease of modernity: Explanations for increasing prevalence. Journal of Affective Disorders, 140(3), 205–214. 10.1016/j.jad.2011.12.036 22244375 PMC3330161

[hbm25193-bib-0033] Kaiser, R. H. , Andrews‐Hanna, J. R. , Wager, T. D. , & Pizzagalli, D. A. (2019). Large‐scale network dysfunction in major depressive disorder a meta‐analysis of resting‐state functional connectivity. JAMA Psychiatry, 02478(6), 603–611. 10.1001/jamapsychiatry.2015.0071 PMC4456260 25785575

[hbm25193-bib-0034] Kapur, S. , Phillips, A. G. , & Insel, T. R. (2012). Why has it taken so long for biological psychiatry to develop clinical tests and what to do about it? Molecular Psychiatry, 17(12), 1174–1179. 10.1038/mp.2012.105 22869033

[hbm25193-bib-0035] Kelly, M. M. , Tyrka, A. R. , Anderson, G. M. , Price, L. H. , & Carpenter, L. L. (2008). Sex differences in emotional and physiological responses to the Trier Social Stress Test. Journal of Behavior Therapy and Experimental Psychiatry, 39, 87–98. 10.1016/j.jbtep.2007.02.003 17466262 PMC4467692

[hbm25193-bib-0036] Kirschbaum, C. , & Hellhammer, D. (1993). The ‘Trier Social Stress Test’—A tool for investigating psychobiological stress responses in a laboratory setting. Neuropsychobiology, 28, 76–81. 10.1159/000119004 8255414

[hbm25193-bib-0037] Kirschbaum, C. , Wust, S. , & Hellhammer, D. (1992). Consistent sex differences in cortisol responses to psychological stress. Psychosomatic Medicine, 657, 648–657. 10.1097/00006842-199211000-00004 1454958

[hbm25193-bib-0038] Klooster, D. , Vos, I. , Caeyenberghs, K. , Leemans, A. , David, S. , Besseling, R. , … Baeken, C. (2019). Structural connectivity between dorsolateral prefrontal cortex and cingulate cortex predicts clinical response to accelerated iTBS in major depression. Brain Stimulation, 12(2), 449–450. 10.1016/j.brs.2018.12.459

[hbm25193-bib-0039] Lefaucheur, J. , Aleman, A. , Baeken, C. , Benninger, D. H. , Brunelin, J. , Grefkes, C. , … Ziemann, U. (2020). Clinical neurophysiology evidence‐based guidelines on the therapeutic use of repetitive transcranial magnetic stimulation (rTMS): An update (2014–2018). Clinical Neurophysiology, 131(2020), 474–528. 10.1016/j.clinph.2019.11.002 31901449

[hbm25193-bib-0040] Li, B. , Friston, K. , Mody, M. , & Hu, H. L. D. (2018). A brain network model for depression: From symptom understanding to disease intervention. CNS Neuroscience & Therapeutics, 24(11), 1004–1019. 10.1111/cns.12998 29931740 PMC6490158

[hbm25193-bib-0041] Li, C. , Wang, S. , Hirvonen, J. , Hsieh, J. , & Bai, Y. (2010). Antidepressant mechanism of add‐on repetitive transcranial magnetic stimulation in medication‐resistant depression using cerebral glucose metabolism. Journal of Affective Disorders, 127(1–3), 219–229. 10.1016/j.jad.2010.05.028 20598753

[hbm25193-bib-0042] López‐Alonso, V. , Cheeran, B. , Río‐Rodríguez, D. , & Fernández‐del‐Olmo, M. (2014). Inter‐individual variability in response to non‐invasive brain stimulation paradigms. Brain Stimulation, 7(3), 372–380. 10.1016/j.brs.2014.02.004 24630849

[hbm25193-bib-0043] Mayberg, H. S. (2009). Targeted electrode‐based modulation of neural circuits for depression. Journal of Clinical Investigation, 119(4), 717–725. 10.1172/JCI38454 19339763 PMC2662569

[hbm25193-bib-0044] McCormack, H. , de Horne, D. J. L. , & Sheather, S. (1988). Clinical applications of visual analogue scales: A critical review. Psychological Medicine, 18(4), 1007–1019. 10.1017/S0033291700009934 3078045

[hbm25193-bib-0045] Morris, M. C. , Compas, B. E. , & Garber, J. (2012). Relations among posttraumatic stress disorder, comorbid major depression, and HPA function: A systematic review and meta‐analysis. Clinical Psychology Review, 32(4), 301–315. 10.1016/j.cpr.2012.02.002 22459791 PMC3340453

[hbm25193-bib-0046] Oakes, P. , Loukas, M. , Oskouian, R. J. , & Tubbs, S. R. (2017). The neuroanatomy of depression: A review. Clinical Anatomy, 49(August 2016), 44–49. 10.1002/ca.22781 27576673

[hbm25193-bib-0047] Padberg, F. , & George, M. S. (2009). Repetitive transcranial magnetic stimulation of the prefrontal cortex in depression. Experimental Neurology, 219(1), 2–13. 10.1016/j.expneurol.2009.04.020 19409383

[hbm25193-bib-0048] Pariante, C. M. , & Lightman, S. L. (2008). The HPA axis in major depression: Classical theories and new developments. Trends in Neurosciences, 31(9), 464–468. 10.1016/j.tins.2008.06.006 18675469

[hbm25193-bib-0049] Pizzagalli, D. A. (2011). Frontocingulate dysfunction in depression: Toward biomarkers of treatment response. Neuropsychopharmacology, 36, 183–206. 10.1038/npp.2010.166 20861828 PMC3036952

[hbm25193-bib-0050] Pruessner, J. C. , Kirschbaum, C. , Meinlschmid, G. , & Hellhammer, D. H. (2003). Two formulas for computation of the area under the curve represent measures of total hormone concentration versus time‐dependent change. Psychoneuroendocrinology, 28(2003), 916–931. 10.1016/S0306-4530(02)00108-7 12892658

[hbm25193-bib-0051] Pulopulos, M. M. , Baeken, C. , & de Raedt, R. (2020). Hormones and behavior cortisol response to stress: The role of expectancy and anticipatory stress regulation. Hormones and Behavior, 117(November 2018), 104587 10.1016/j.yhbeh.2019.104587 31639385

[hbm25193-bib-0052] Pulopulos, M. M. , de Witte, S. , Vanderhasselt, M. , de Raedt, R. , Schiettecatte, J. , Anckaert, E. , … Baeken, C. (2019). The influence of personality on the effect of iTBS after being stressed on cortisol secretion. PLoS One, 14(10), 1–16. 10.17605/OSF.IO/KTHMV PMC6795454 31618272

[hbm25193-bib-0053] Qin, S. , Hermans, E. J. , van Marle, H. J. F. , Luo, J. , & Fernández, G. (2009). Acute psychological stress reduces working memory‐related activity in the dorsolateral prefrontal cortex. Biological Psychiatry, 66, 25–32. 10.1016/j.biopsych.2009.03.006 19403118

[hbm25193-bib-0054] Ramirez‐mahaluf, J. P. , Perramon, J. , Otal, B. , Villoslada, P. , & Compte, A. (2018). Subgenual anterior cingulate cortex controls sadness‐induced modulations of cognitive and emotional network hubs. Scientific Reports, 8(8566), 1–16. 10.1038/s41598-018-26317-4 29867204 PMC5986810

[hbm25193-bib-0055] Rijpkema, M. , Smidts, A. , Klucharev, V. , & Hyto, K. (2009). Reinforcement learning signal predicts social conformity. Neuron, 61, 140–151. 10.1016/j.neuron.2008.11.027 19146819

[hbm25193-bib-0056] Rogers, M. A. , Kasai, K. , Koji, M. , Fukuda, R. , Iwanami, A. , Nakagome, K. , … Kato, N. (2004). Executive and prefrontal dysfunction in unipolar depression: A review of neuropsychological and imaging evidence. Neuroscience Research, 50, 1–11. 10.1016/j.neures.2004.05.003 15288493

[hbm25193-bib-0057] Seeley, W. W. , Menon, V. , Schatzberg, A. F. , Keller, J. , Glover, G. H. , Kenna, H. , … Greicius, M. D. (2007). Dissociable intrinsic connectivity networks for salience processing and executive control. Journal of Neuroscience, 27(9), 2349–2356. 10.1523/JNEUROSCI.5587-06.2007 17329432 PMC2680293

[hbm25193-bib-0069] Sheehan, D. V. , Lecrubier, Y. , Sheehan, K. H. , Amorim, P. , Janavs, J. , Weiller, E. , & Dunbar, G. C. (1998). The Mini‐International Neuropsychiatric Interview (M.I.N.I): The development and validation of a structured diagnostic psychiatric interview for DSM‐IV and ICD‐10. The Journal of Clinical Psychiatry, 59(Suppl 20), 22–33. 9881538

[hbm25193-bib-0058] Silverstein, W. K. , Sc, B. M. , Noda, Y. , Ph, D. , Barr, M. S. , Sc, M. , & Ph, D. (2015). Neurobiological predictors of response to dorsolateral prefrontal cortex repetitive transcranial magnetic stimulation in depression: A systematic review. Depression and Anxiety, 32(September), 871–891. 10.1002/da.22424 26382227

[hbm25193-bib-0059] Stetler, C. , & Miller, G. E. (2011). Depression and hypothalamic‐pituitary‐adrenal activation: A quantitative summary of four decades of research. Journal of Biobehavioral Medicine, 126, 114–126. 10.1097/PSY.0b013e31820ad12b 21257974

[hbm25193-bib-0060] Tik, M. , Sladky, R. , Tomova, L. , Hummer, A. , Navarro, L. , Lara, D. , … Windischberger, C. (2017). Towards understanding rTMS mechanism of action: Stimulation of the DLPFC causes network‐specific increase in functional connectivity. NeuroImage, 162(September), 289–296. 10.1016/j.neuroimage.2017.09.022 28912081

[hbm25193-bib-0061] van der Does, A. J. W. (2002). BDI‐II‐NL. Handleiding In De Nederlandse versie van de Beck Depression Inventory (2nd ed.). Lisse: Harcourt Test Publishers.

[hbm25193-bib-0062] Wang, Y. , Chen, G. , Zhong, S. , Jia, Y. , Xia, L. , & Lai, S. (2018). Association between resting‐state brain functional connectivity and cortisol levels in unmedicated major depressive disorder. Journal of Psychiatric Research, 105(August), 55–62. 10.1016/j.jpsychires.2018.08.025 30189325

[hbm25193-bib-0063] Wang, Y. , Yang, S. , Sun, W. , Shi, Y. , & Duan, H. (2016). Altered functional interaction hub between affective network and cognitive control network in patients with major depressive disorder. Human Brain Mapping, 298, 301–309. 10.1016/j.bbr.2015.10.040 26519557

[hbm25193-bib-0064] Weigand, A. , Horn, A. , Caballero, R. , Cooke, D. , Stern, A. P. , Taylor, S. F. , … Fox, M. D. (2018). Prospective validation that subgenual connectivity predicts antidepressant efficacy of transcranial magnetic stimulation sites. Biological Psychiatry, 84(1), 28–37. 10.1016/j.biopsych.2017.10.028 29274805 PMC6091227

[hbm25193-bib-0068] Whitfield‐Gabrieli, S. , & Nieto‐Castanon, A. (2012). Conn: A functional connectivity toolbox for correlated and anticorrelated brain networks. Brain Connectivity, 2, 125–141. 10.1089/brain.2012.0073 22642651

[hbm25193-bib-0065] Xia, M. , Wang, J. , & He, Y. (2013). BrainNet viewer: A network visualization tool for Human Brain Connectomics. PLoS One, 8(7), e68910 10.1371/journal.pone.0068910 23861951 PMC3701683

[hbm25193-bib-0066] Ycaza, A. , Faude, S. , Nielsen, S. E. , Locke, M. , & Mather, M. (2019). Effects of hormonal contraceptive phase and progestin generation on stress‐induced cortisol and progesterone release. Neurobiology of Stress, 10(February), 100151 10.1016/j.ynstr.2019.100151 30937356 PMC6430619

