# Supplementary material for: Individual resting‐state frontocingular functional connectivity predicts the intermittent theta burst stimulation response to stress in healthy female volunteers
Source: Hum Brain Mapp. 2020 Oct 3;41(18):5301–12. doi: 10.1002/hbm.25193 (PMC7670632; doi:10.1002/hbm.25193)
Supplement: Supplementary file 1 — Appendix S1: Supplementary materials [file HBM-41-5301-s001.docx]

1. **Supplementary materials**
2. **MNI coordinates of ROI’s**

| ***Regions of interest*** | ***MNI coordinates*** | | |
| --- | --- | --- | --- |
|  | *X* | *Y* | *Z* |
| *Dorsolateral prefrontal cortex (DLPFC)* | *-39.74 22.65 53.54* | | |
| *Caudal ACC (cACC)* | *(-)5* | *-10* | *47* |
| *Dorsal ACC (dACC)* | *(-)5* | *14* | *42* |
| *Rostral ACC (rACC)* | *(-)5* | *34* | *28* |
| *Perigenual ACC (pACC)* | *(-)5* | *47* | *11* |
| *Subgenual ACC (sACC)* | *(-)5* | *25* | *-10* |

1. **Summary of the results controlling for time of the day:**

The results of the mixed ANOVA with AUCi and AUCg as dependent variables and time of the day as covariate showed no significant main effect of Stimulation (AUCi: F(1,31)=0.92, p=.344; AUCg: F(1,31)=0.03, p=.871), Order (AUCi: F(1,31)=0.64, p=.429; AUCg: F(1,31)=1.22, p=.279), or the interaction between Stimulation and Order (AUCi: F(1,31)=0.87, p=.358; AUCg: F(1,31)=0.46, p=.504). These results indicate that iTBS does not affect AUCi and AUCg after being stressed. Time of the day was significant for AUCg (F(1,31)=4.29, p=.047), but not for AUCi (F(1,31)=0.82, p=.373). The interaction between time of the day and Stimulation were not statistically significant (AUCi: F(1,31)=0.80, p=.379; AUCg: F(1,31)=0.03, p=.858).

Independent mixed ANCOVAs with time of the day and each rsFC index as covariates were performed to investigate the influence of rsFC between the DLPFC and the subparts of ACC on the effects of iTBS on the activity of the HPA axis after stress. For AUCi, the results showed a no significant effect of Stimulation, Order, time of the day, and their interactions (F(1,30)<1.60, p>.217). For AUCg, the effect of Stimulation, Order, their interaction, and the interaction with time of the day were not statistically significant (F(1,30)<1.31, p>.261). (F(1,31)<0.73, p>.400). The factor time of the day showed p>0.044. These results are not considered statistically significant after a correction for multiple comparisons is applied (only p<0.0025 are considered statistically significant). None of the rsFC indexes showed a significant main effect (AUCi: F(1,30)<2.53, p>.122; AUCg: F(1,30)<0.63, p>.453).

Regarding the influence of the rsFC on the effects of iTBS on the stress response, we observed a significant rsFC interaction between the left DLPFC and the left cACC and the factor Stimulation for AUCi (F(1,30)=13.24, p=.001), but not for AUCg (F(1,30)=4.06, p=.053). None of the other interactions between rsFC indexes and Stimulation showed a significant effect (AUCi: F(1,30)<1.46, p>.236; AUCg: F(1,30)<2.79, p>.105).

Finally, correlation analyses were performed to further investigate the meaning of the significant interaction between Stimulation and the rsFC between the left DLPFC and the left cACC. The results of the correlation analysis showed a significant rsFC association between left cACC-DLPFC and the AUCi during the active iTBS session (r=0.531, p=.002), but not during the sham iTBS session (r=-0.187, p=.299). These results indicate that the weaker the rsFC between the left DLPFC and the left cACC, the lower the AUCi during active-iTBS, but not during sham-iTBS.
